# Use of Multimodal Artificial Intelligence in Surgical Instrument Recognition

**DOI:** 10.3390/bioengineering12010072

**Published:** 2025-01-15

**Authors:** Syed Ali Haider, Olivia A. Ho, Sahar Borna, Cesar A. Gomez-Cabello, Sophia M. Pressman, Dave Cole, Ajai Sehgal, Bradley C. Leibovich, Antonio Jorge Forte

**Affiliations:** 1 Division of Plastic Surgery, Mayo Clinic, Jacksonville, FL 32224, USA; 2 Center for Digital Health, Mayo Clinic, Rochester, MN 55905, USA; 3 Department of Urology, Mayo Clinic, Rochester, MN 55905, USA

**Keywords:** artificial intelligence, AI, surgical instrument, multimodal AI, computer vision

## Abstract

Accurate identification of surgical instruments is crucial for efficient workflows and patient safety within the operating room, particularly in preventing complications such as retained surgical instruments. Artificial Intelligence (AI) models have shown the potential to automate this process. This study evaluates the accuracy of publicly available Large Language Models (LLMs)—ChatGPT-4, ChatGPT-4o, and Gemini—and a specialized commercial mobile application, Surgical-Instrument Directory (SID 2.0), in identifying surgical instruments from images. The study utilized a dataset of 92 high-resolution images of 25 surgical instruments (retractors, forceps, scissors, and trocars) photographed from multiple angles. Model performance was evaluated using accuracy, weighted precision, recall, and F1 score. ChatGPT-4o exhibited the highest accuracy (89.1%) in categorizing instruments (e.g., scissors, forceps). SID 2.0 (77.2%) and ChatGPT-4 (76.1%) achieved comparable accuracy, while Gemini (44.6%) demonstrated lower accuracy in this task. For precise subtype identification of instrument names (like “Mayo scissors” or “Kelly forceps”), all models had low accuracy, with SID 2.0 having an accuracy of 39.1%, followed by ChatGPT-4o (33.69%). Subgroup analysis revealed ChatGPT-4 and 4o recognized trocars in all instances. Similarly, Gemini identified surgical scissors in all instances. In conclusion, publicly available LLMs can reliably identify surgical instruments at the category level, with ChatGPT-4o demonstrating an overall edge. However, precise subtype identification remains a challenge for all models. These findings highlight the potential of AI-driven solutions to enhance surgical-instrument management and underscore the need for further refinements to improve accuracy and support patient safety.

## 1. Introduction

Artificial intelligence (AI) is quickly infiltrating the healthcare sector. AI is set to transform surgical care akin to past medical breakthroughs like anesthesia, antibiotics, and Minimally Invasive Surgery (MIS). Operating Rooms (ORs) are known for their high-risk nature and dependency on advanced technology [1,2]. AI offers diverse uses within the field of surgery, including tasks like predicting procedure duration, analyzing video data, identifying surgical workflow patterns, assisting with endoscopic navigation, and tracking bone movement [3,4,5,6,7]. The potential of AI to significantly enhance surgical procedures and operational efficiency indicates a promising avenue for application. As operating rooms become increasingly equipped with sensors, video technology, and other hardware, they produce a wealth of data [8,9,10]. The data generated during surgery offers a wealth of information that AI can leverage to drive improvements and generate valuable insights.

Given the unique challenges in the OR, such as the handling and management of instruments, where AI could offer a significant solution, this potential is substantial. The manual identification of surgical instruments within the OR significantly impacts the efficiency and safety of surgical procedures. This labor-intensive process is fraught with the potential for human error. Instruments can be easily miscounted, misplaced, or incorrectly identified, leading to delays and potential hazards, including the dire risk of Retained Surgical Instruments (RSI) [11,12,13]. The variety of instruments used exacerbates these challenges. Staff are expected to recognize and differentiate among hundreds of tools under the timed-pressure and high-stakes surgical setting. This necessitates extensive training and expertise, yet still does not eliminate the risk of errors, as the process is inherently error-prone due to human involvement. Relying on manual instrument tracking creates two significant risks. First, increased handling can lead to contamination, jeopardizing infection control efforts [14]. Second, manual tracking makes it difficult to maintain accurate documentation of the list of instruments.

Moreover, a surgeon’s preference for customized instrument trays adds another layer of complexity. Each surgeon may use a distinct set of instruments, and over time, these trays undergo modifications to suit their specific techniques and requirements better. This evolution of surgical trays makes it particularly challenging to maintain an updated record of which instruments are used more frequently and which are seldom needed [15]. The lack of precise tracking leads to difficulties in optimizing inventory, resulting in potential wastage of resources and inefficiencies in surgical workflow. Rarely used instruments still need to be sterilized, maintained, and stored, consuming valuable resources without contributing to surgical outcomes [16]. Conversely, frequently used instruments may not be available in the required quantities or wear out more quickly without proper tracking and management [17]. A practical solution that could monitor and analyze the usage frequency of each instrument would significantly enhance operational efficiency. By identifying which instruments are essential and which are superfluous, hospitals could tailor their inventories more closely to actual needs, reducing wastage and ensuring that surgical teams have ready access to the tools they require most [18]. Such a system would streamline the surgical workflow, providing more efficient, cost-effective, and high-quality patient care. These challenges with the current process underline the urgent need for an automated solution.

AI allows for automating and streamlining surgical-instrument detection [19]. Its cost-effectiveness and cloud-based accessibility democratize the process, making it viable even in remote or resource-limited settings. This proof-of-concept study aims to assess the capabilities of AI systems, mainly publicly available Large Language Models (LLMs), for surgical-instrument recognition without using the Retrieval-Augmented Generation (RAG) process. This study introduces a novel approach to surgical-instrument recognition by evaluating the capabilities of publicly available LLMs in this domain. While previous research has focused on developing specialized computer-vision models and custom AI solutions for surgical-instrument detection, our study is the first to assess how readily available mobile LLMs like ChatGPT and Gemini perform in this task compared to specialized commercial mobile applications. This approach is particularly significant as it explores whether widely accessible AI tools could provide a more cost-effective and scalable alternative to traditional hardware-based or custom-developed solutions. Our paper first provides a comprehensive overview of AI models’ current state and challenges in surgical-instrument recognition, emphasizing both public and specialized systems. We then detail our methodology of testing four AI models (ChatGPT-4, ChatGPT-4o, Gemini, and SID 2.0) on a dataset of 92 surgical-instrument images, then presenting comparative results and performance metrics. The paper concludes with an in-depth discussion of our findings, practical applications, limitations, and future implications for AI-driven surgical-instrument recognition in healthcare settings.

## 2. Literature Review

Research in surgical-instrument detection has evolved significantly, with particular emphasis on applications in Minimally Invasive Surgery (MIS) and patient safety enhancement [20,21]. Recent advances have produced two primary methodological approaches: vision-based and hardware-based detection systems [22], each addressing different aspects of surgical-instrument management. Vision-based technologies utilize computer vision and AI to analyze imagery from surgical cameras or endoscopes [23,24]. The effectiveness of these systems has been demonstrated by Deol et al., who developed a deep learning-based computer-vision model achieving remarkable precision (98.5%) and recall (99.9%) in distinguishing surgical tools, even maintaining this performance with overlapping instruments [25]. This approach can employ feature extraction or more advanced deep learning methods. The advantage of vision-based technologies is that they can easily integrate with existing surgical setups and are low-cost because they do not need additional hardware. Wagner et al. showed that combining imaging data with other operating room information in a knowledge graph-based approach achieved a 66.86% F1 score in instrument anticipation tasks, illustrating how comprehensive data integration can enhance surgical workflow prediction [26].

Other significant contributions include Funke et al.’s innovative deep learning method using an inflated 3D Convolutional Neural Network (ConvNet) [27] and Lavanchy et al.’s sophisticated three-stage machine learning system for evaluating laparoscopic cholecystectomy [28]. Subsequent stages involve the extraction of motion features and linear regression model application, designed to predict a surgeon’s skill level based on their movements. The evolution of CNN models, particularly YOLOv7x with its Effective Layer Aggregated Network (E-LAN) architecture, has significantly advanced instrument detection capabilities [16]. The M2CAI 2016 Tool Presence Detection Challenge established new benchmarks for surgical tool detection [29], while Google’s SAVI (Semi-Automated Vision Inspection) system specifically addresses the critical need for thorough inspection of surgical trays [30].

While vision-based systems offer advantages such as easy integration with existing surgical setups and low-cost implementation, they face challenges, including the need for manually labeled training data and sensitivity to visual occlusions and lighting variations [31]. In contrast, hardware-based solutions employ RFID tags, Electromagnetic (EM) tracking, and Optical Tracking systems, requiring physical modifications to either the surgical instruments or the operating room environment [32]. Building upon this foundation, our research explores a novel application of publicly available LLMs for surgical-instrument recognition. While these models represent another computer-vision technology, their use in surgical settings is unique. They offer the advantage of not requiring specialized training or custom datasets, potentially making automated instrument recognition systems more accessible in resource-limited settings. Our study’s core problem is the challenge of accurate, efficient, and cost-effective surgical-instrument identification in operating rooms. Manual identification is time-consuming, error-prone, and carries risks of Retained Surgical Items. While specialized hardware solutions exist, they are often expensive and require infrastructure changes. Our study investigates whether publicly available AI models could offer an accessible alternative.

## 3. Methods

### 3.1. Dataset Creation

We assembled a dataset of 92 high-resolution images that captured 25 distinct types of surgical instruments, categorized into four main groups. The first category, retractors, included 7 varieties: the Malleable Retractor, Army Navy Retractor, Daever Retractor, Rake Retractor, Richardson Retractor, Senn Retractor, and Weitlaner Retractor. The second and largest category comprised 13 different types of forceps: Babcock, Kocher, Crile, Adson Brown, Allis, DeBakey, Dressing, Gerald, Mosquito, Rat Tooth, Sponge, Kelly, and Tissue Forceps. The third category consisted of 4 types of scissors: Bandage, Iris, Mayo, and Tenotomy Scissors. The fourth and final category contained a single instrument type: the trocar. This diverse collection of surgical instruments represented standard tools used in various surgical procedures, providing a robust foundation for testing the AI models’ recognition capabilities.

### 3.2. AI Models Assessed

This study leveraged publicly available LLMs that did not employ RAG techniques. Additionally, a commercially available application for surgical-instrument recognition was utilized. The details of the AI models are below:

1. ChatGPT-4: A generative pre-trained transformer model developed by OpenAI [33].
2. ChatGPT-4o (aka omni): The multimodal version of ChatGPT by OpenAI that is specially optimized for visual analysis, object identification, audio recognition, and translation tasks [34].
3. Gemini: Google DeepMind developed Gemini (previously known as Bard), a suite of Large Language Models building upon the successes of previous models like LaMDA and PaLM 2 [35]. Gemini is designed to be multimodal, seamlessly integrating its understanding of text, code, audio, images, and video.
4. SID 2.0: The Surgical-Instrument Directory (SID) is a commercially available web/mobile-based AI application designed by LayerJot specifically for surgical-instrument identification [36]. It is trained on a database of over 4 million instruments. Its features include image recognition, barcode scanning, and a searchable database.

### 3.3. Image Acquisition and Evaluation

**Figure 1:**
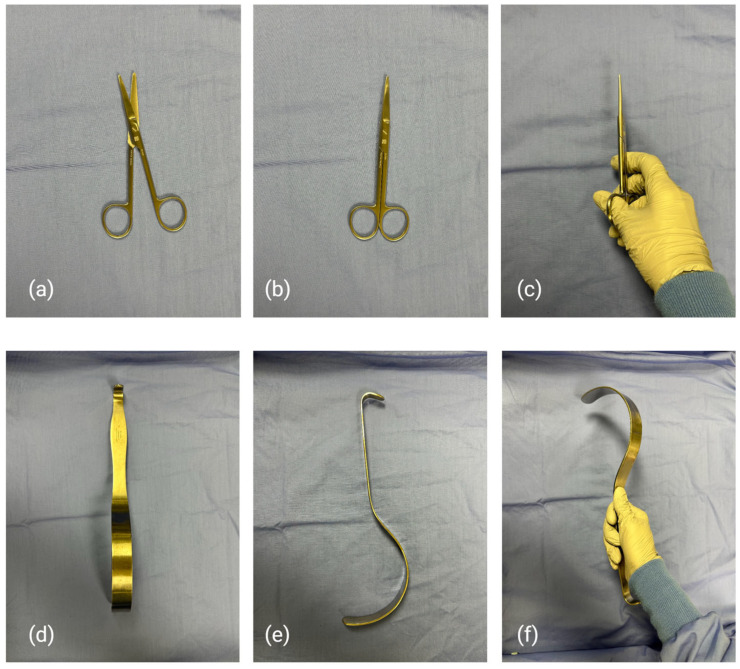
(**a**–**c**) Image of Mayo Scissors taken from different angles (**d**–**f**) Image of Deavers Retractor taken from different angles.

Images were captured from multiple perspectives to simulate real-world surgical environments. Instruments were shown both resting on the blue surgical drapes and held in hand wearing surgical gloves (Figure 1). All images were taken by an iPhone 14 Pro.

**Figure 2:**
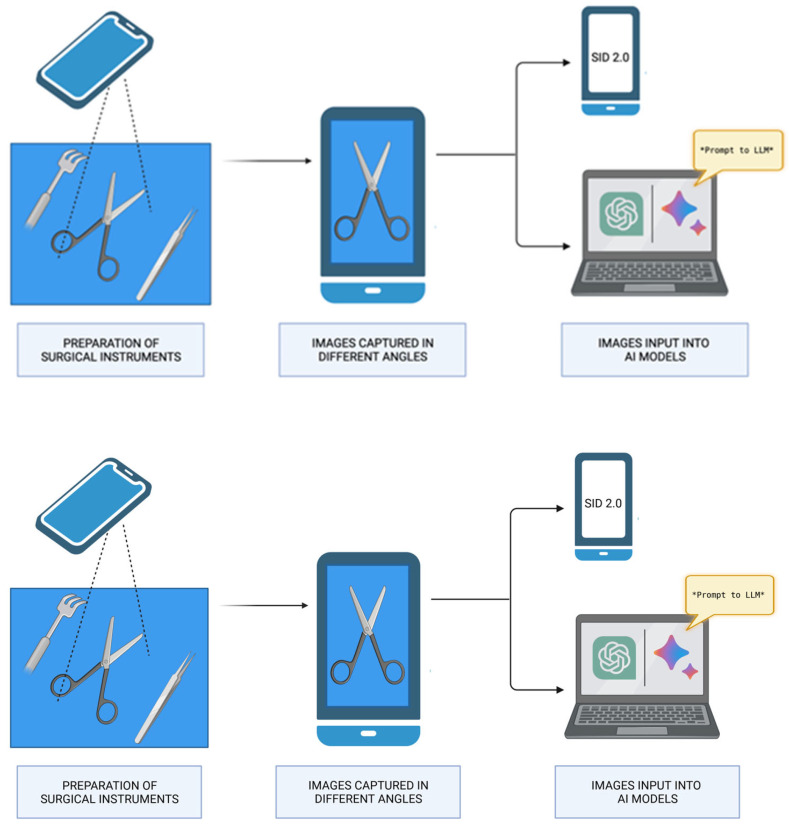
Image Acquisition and Evaluation. Created with BioRender© (https://www.biorender.com/, accessed on 13 January 2025).

For the LLMs, a standard prompt was used (Figure 2):

“I am providing you with a picture of a surgical instrument. Please do your best to identify the following:

i. The Category of the instrument:
ii. The Specific Name of the instrument:”

### 3.4. Analysis

A comprehensive evaluation was conducted across the four AI models using 92 test images, generating 736 distinct classification attempts (92 images × 4 models × 2 classification tasks). Each model was assessed on its ability to identify both the general instrument category and the specific instrument name, creating a two-tiered evaluation framework. The performance metrics, such as accuracy, weighted precision, recall, and F1 scores, were calculated for each model to provide an assessment of their classification capabilities. Subgroup analysis was also performed to examine performance variations across different instrument categories.

## 4. Results

**Figure 3:**
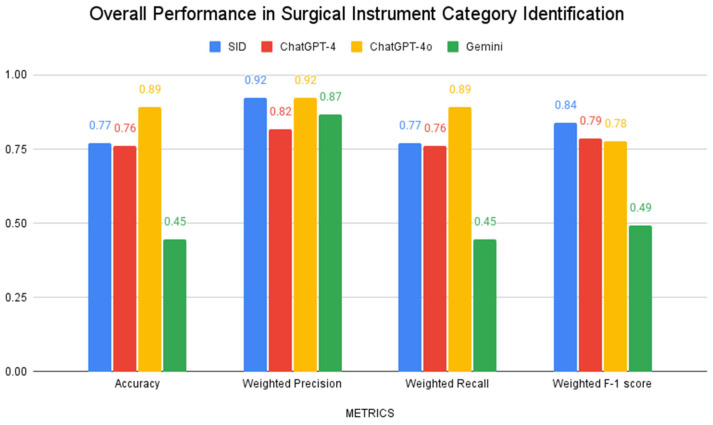
Model performance across various surgical-instrument category identification tasks as demonstrated by accuracy, weighted precision, weighted recall, and weighted F-1 score.

Performance analysis across all four AI models showed varying capabilities in surgical-instrument identification tasks. For general instrument categories (e.g., “scissors”, “forceps”), ChatGPT-4o achieved the highest accuracy (89%), while both SID and ChatGPT-4 demonstrated similar accuracy (77% and 76%), and Gemini had the lowest accuracy at 45%. SID achieved the highest weighted F-1 score (0.84), followed by ChatGPT-4 (0.79) and ChatGPT-4o (0.78), with Gemini showing notably lower performance across all metrics. Performance metrics for general categorization are shown in Figure 3.

**Figure 4:**
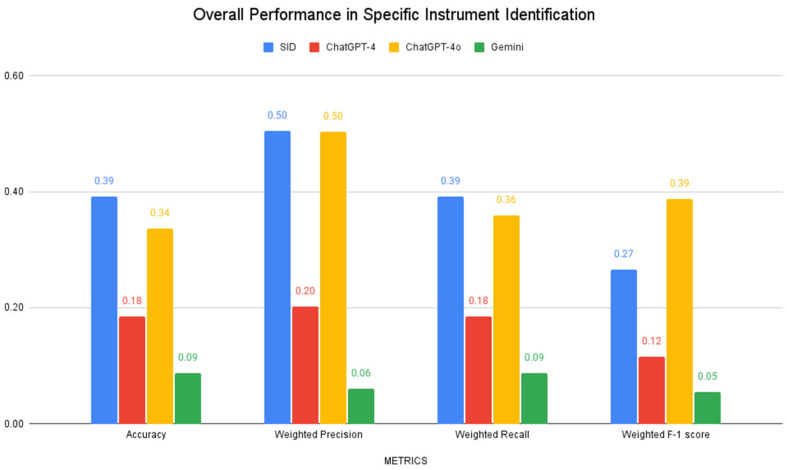
Model performance in surgical-instrument subtype identification, including accuracy, weighted precision, weighted recall, and weighted F1 score.

In specific instrument-subtype classification (e.g., “Mayo scissors”, “Kelly forceps”), all models showed substantially lower performance. SID achieved the highest accuracy (39%), while ChatGPT-4o demonstrated the highest weighted F-1 score (0.39). Both models shared equal weighted precision (0.50), though ChatGPT-4 and Gemini showed markedly lower performance across all metrics, as illustrated in Figure 4. Detailed classification patterns and error types for each model are presented in confusion matrices in Table 1, Table 2, Table 3 and Table 4.

### Subgroup Analysis

**Figure 5:**
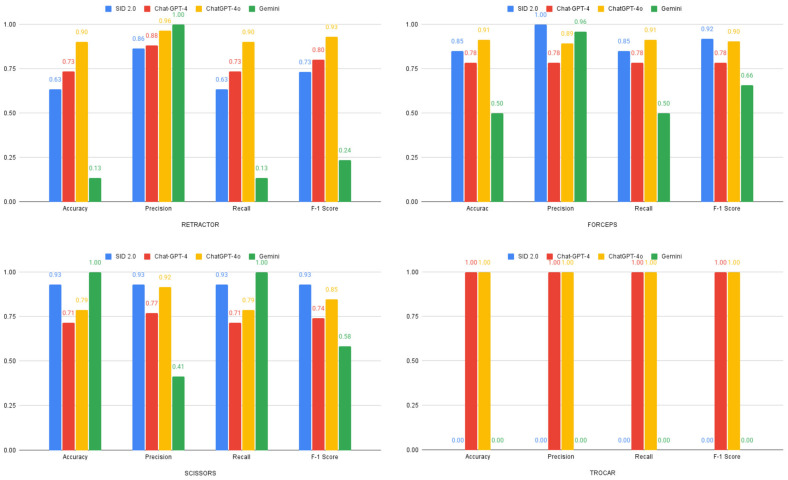
Model performance across various surgical-instrument category identification tasks as demonstrated by accuracy, precision, recall, and F-1 score.

Analysis by instrument category revealed varying strengths across models, as shown in Figure 5.

## 5. Discussion

The findings in this study highlight substantial variability in the performance of four AI models—ChatGPT-4o, ChatGPT-4, SID2.0, and Gemini—when identifying both surgical-instrument categories and specific instrument names. For category-level identification, ChatGPT-4o outperformed all other models with an accuracy of 89.1%, followed by SID2.0 (77.2%) and ChatGPT-4 (76.1%) with similar accuracy levels, while Gemini trailed at 44.6%. These results suggest that ChatGPT-4o is the most robust choice for categorizing instruments into general groups (e.g., scissors, forceps, trocars, retractors).

However, when identifying precise instrument names, all models demonstrated a dramatic drop in performance. SID led this specific-level classification with an accuracy of 39%, while ChatGPT-4o achieved 34% accuracy and the highest weighted F1-score of 0.39. Both models achieved equal weighted precision (0.50), suggesting similar confidence in their positive predictions. ChatGPT-4 (18.5% accuracy) and Gemini (9% accuracy) showed markedly lower performance in specific instrument identification. These significant performance drops between general and specific identification (e.g., from 89% to 34% for ChatGPT-4o) highlight a critical limitation in current AI systems’ ability to make fine-grained distinctions between similar surgical instruments.

### 5.1. Model Performance Insights

Analysis of performance by instrument category reveals distinct patterns and challenges across the four models. The varying accuracy and reliability observed in this study can be attributed to several factors. First, dataset quality and diversity play a critical role, as models trained on larger, more heterogeneous image sets tend to capture better the nuances of shapes, textures, and reflective properties inherent to surgical instruments. SID2.0, for instance, has reportedly trained on millions of instrument images, enhancing its ability to identify subtle differences in instrument design. Conversely, language-based models often rely on broader, less specialized training data. Notably, ChatGPT-4o was developed with enhanced image-recognition capabilities and is optimized for general identification tasks, which likely explains its strong performance in categorizing instruments, even if it struggled with more precise naming. Second, image quality and context—including lighting, angle, and resolution—can shape model output. Even minor differences in image characteristics may influence the final predictions, especially when instruments have similar shapes or features. Finally, instrument variability remains a significant challenge: retractors, for example, exhibit considerable diversity in size, shape, and design, and instruments less frequently represented in training sets tend to be recognized at lower rates. These factors, taken together, underscore the importance of specialized training data and robust, multimodal approaches for more accurate AI-driven surgical-instrument recognition.

### 5.2. Practical Applications

Despite these performance differentials, all four models share notable advantages: they can be accessed via a simple smartphone application and require minimal hardware. Such accessibility is invaluable in resource-limited settings. Furthermore, deploying an automated instrument recognition tool can enhance patient safety by reducing the risk of RSI. By keeping an accurate count of instruments, AI solutions address one of the critical safety concerns in the OR [37,38]. Integrating AI in perioperative workflows can streamline instrument setup, sterilization, and post-operative processing. By automating these labor-intensive tasks, OR teams can focus on high-value patient care activities. Beyond the OR, AI can power inventory control and surgical set assembly, potentially decreasing costs and reducing human error [39,40]. As these systems become more sophisticated, LLMs could also analyze usage patterns to recommend optimized tray configurations, aiding in cost-reduction strategies and more efficient staffing.

While specialized models like SID2.0 are trained to solely recognize instruments, Multimodal LLMs such as ChatGPT-4o offer a breadth of contextual and analytical capabilities that could further enhance decision-making in surgery. Multimodal AI—integrating text, images, voice, and potentially other sensor data—could resolve ambiguities and bolster identification and classification tasks [41]. This synergy might enable voice-driven queries (“Identify that clamp”, “Is this a Kelly forceps”?) with real-time validation by a visual AI subsystem. Nevertheless, designing effective multimodal architectures remains an open area of research, requiring careful coordination of data streams and robust model training.

Existing surgical skill assessment platforms could benefit from integrating AI-driven insights on instrument usage, therefore providing objective feedback on surgeons’ technique and efficiency [22]. In parallel, it is critical to develop and maintain a comprehensive, well-annotated database of surgical instruments to ensure accurate detection across a wide array of clinical scenarios. Rigorous data security measures must remain a priority to protect patient and institutional confidentiality.

The contrast between category-level and specific instrument identification capabilities has important implications for practical implementation. While the high accuracy in general categorization (particularly by ChatGPT-4o) suggests potential utility in basic instrument tracking and inventory management, the significant drop in performance for specific instrument identification indicates current limitations for more precise applications. This distinction is crucial for healthcare settings where accurate instrument identification is vital for patient safety and procedural efficiency. LLMs in their current state should not be relied on for instrument identification. Developing comprehensive, well-annotated datasets representing the full spectrum of surgical instruments in various conditions is imperative. These datasets will be the bedrock upon which robust and adaptable AI models can differentiate between instruments’ conditions. Moreover, a seamless integration with existing systems is paramount for smooth hospital adoption. Finally, the responsible handling of sensitive data generated by these systems is critical. Data security and privacy protocols must always be in place to ensure AI’s ethical and secure use in the surgical setting.

### 5.3. Limitations

The primary methodological challenge in our study stemmed from the varying image-processing capabilities across the evaluated models. While ChatGPT can process multiple images simultaneously, Gemini is limited to single-image analysis, and SID 2.0 is designed explicitly for two-image comparison. To maintain consistency in our evaluation, we implemented a single-image approach where ChatGPT and Gemini received one image, while SID 2.0 was provided with duplicate copies of the same image. We recognize that this standardization may not have utilized each model’s full capabilities.

Our dataset included 92 images from 25 surgical instruments in this proof-of-concept study. While this served as an effective testing dataset for pre-trained models, certain instrument categories, particularly trocars, had a smaller representation. Future research would benefit from testing with a larger variety of surgical tools to validate the performance patterns observed, especially for underrepresented instrument categories.

Furthermore, while our study attempted to simulate a surgical environment, the simplified nature of our image dataset did not fully capture the complexity of real-world operating rooms. In actual surgical scenarios, instruments are rarely presented in isolation. Instead, they appear in dynamic environments with multiple complications: tools may be partially obscured, lighting conditions can vary significantly, surrounding tissue/blood is often present, and various instruments move simultaneously. These real-world factors introduce additional challenges that our current image dataset has not addressed.

## 6. Conclusions

This study demonstrates that publicly available LLMs can effectively categorize surgical instruments, with ChatGPT-4o achieving 89% accuracy (precision = 0.92) in broad classification tasks. However, specific instrument identification remains challenging, with specialized SID 2.0 reaching the highest accuracy of only 39%. The performance varied significantly across instrument types—both ChatGPT-4 and ChatGPT-4o achieved perfect recognition of trocar (100% accuracy), while Gemini achieved 100% accuracy for the scissor category but with low precision (0.41), and SID 2.0 demonstrated a more balanced performance (77% accuracy and 0.92 precision).

These findings fill critical knowledge gaps by providing the first direct comparison between general-purpose LLMs and specialized medical applications in surgical-instrument recognition. The study reveals that accessible, low-cost AI solutions using public LLMs can match or exceed specialized mobile applications for basic categorization tasks, though precise instrument identification still requires improvement. This creates a foundation for developing more accurate and cost-effective surgical-instrument recognition systems that could enhance operating room efficiency and patient safety, particularly in resource-limited settings.

Future research should focus on improving specific instrument recognition capabilities and exploring hybrid approaches that combine the strengths of both general-purpose LLMs and specialized medical applications. A key direction would be testing the multimodal abilities of LLMs by incorporating audio, real-time operating room images, and surgical videos. Additionally, expanding the test dataset beyond the current 25 instruments would provide more comprehensive performance metrics across a broader range of surgical tools. Future experimentation should also investigate specialized RAG techniques to enhance the accuracy of surgical-instrument identification, particularly for rare or specialized tools.

## Figures and Tables

**Table 1:** Confusion Matrix representing the performance of SID 2.0 in identifying the category of the surgical instrument.

SID 2.0		Actual					
		Retractor	Forceps	Scissor	Trocar	Other	NA
Predicted	Retractor	19	2	0	1	0	0
	Forceps	0	39	0	0	0	0
	Scissor	0	1	13	0	0	0
	Trocar	0	0	0	0	0	0
	Other	0	3	1	1	0	0
	NA	11	1	0	0	0	0

**Table 2:** Confusion Matrix representing the performance of ChatGPT-4 in identifying the category of the surgical instrument.

ChatGPT-4		Actual					
		Retractor	Forceps	Scissor	Trocar	Other	NA
Predicted	Retractor	22	3	0	0	0	0
	Forceps	6	36	4	0	0	0
	Scissor	0	3	10	0	0	0
	Trocar	0	0	0	2	0	0
	Other	2	4	0	0	0	0
	NA	0	0	0	0	0	0

**Table 3:** Confusion Matrix representing the performance of Gemini in identifying the category of the surgical instrument.

Gemini		Actual					
		Retractor	Forceps	Scissor	Trocar	Other	NA
Predicted	Retractor	4	0	0	0	0	0
	Forceps	1	23	0	0	0	0
	Scissor	3	17	14	0	0	0
	Trocar	0	0	0	0	0	0
	Other	12	6	0	0	0	0
	NA	10	0	0	2	0	0

**Table 4:** Confusion Matrix representing the performance of ChatGPT-4o in identifying the category of the surgical instrument.

ChatGPT 4o		Actual					
		Retractor	Forceps	Scissor	Trocar	Other	NA
Predicted	Retractor	27	1	0	0	0	0
	Forceps	2	42	3	0	0	0
	Scissor	0	1	11	0	0	0
	Trocar	0	0	0	2	0	0
	Other	1	2	0	0	0	0
	NA	0	0	0	0	0	0

## Data Availability

The raw data supporting the conclusions of this article will be made available by the authors upon request.

## Abbreviations

AI: Artificial Intelligence; SID, Surgical-Instrument Directory; RAG, Retrieval-Augmented Generation.

## References

[1] Schimpff S.C. (2007). Improving Operating Room and Perioperative Safety: Background and Specific Recommendations. Surg. Innov..

[2] Schurr M.O., Buess G.F. (2000). Systems technology in the operating theatre: A prerequisite for the use of advanced devices in surgery. Minim. Invasive Ther. Allied Technol..

[3] Palen T., Tavel H., Brill J., Bajaj J. (2014). B1-1: Predictive Modeling to Identify Patients at Risk for Index Hospitalization. Clin. Med. Res..

[4] ShahabiKargar Z., Khanna S., Good N., Sattar A., Lind J., O’Dwyer J. (2014). Predicting Procedure Duration to Improve Scheduling of Elective Surgery. PRICAI 2014: Trends in Artificial Intelligence: 13th Pacific Rim International Conference on Artificial Intelligence, Gold Coast, QLD, Australia, 1–5 December 2014.

[5] Jiao Y., Sharma A., Ben Abdallah A., Maddox T.M., Kannampallil T. (2020). Probabilistic forecasting of surgical case duration using machine learning: Model development and validation. J. Am. Med. Inform. Assoc..

[6] Messmann H. (2021). Artificial Intelligence in Endoscopy. Bildverarb. Med..

[7] Liu H., Baena F.R.Y. (2020). Automatic Markerless Registration and Tracking of the Bone for Computer-Assisted Orthopaedic Surgery. IEEE Access.

[8] Kawka M., Gall T.M.H., Fang C., Liu R., Jiao L.R. (2021). Intraoperative video analysis and machine learning models will change the future of surgical training. Intell. Surg..

[9] Padoy N. (2019). Machine and deep learning for workflow recognition during surgery. Minim. Invasive Ther. Allied Technol..

[10] Ward T.M., Mascagni P., Ban Y., Rosman G., Padoy N., Meireles O.R., Hashimoto D.A. (2020). Computer vision in surgery. Surgery.

[11] Johnson A. (2021). Manual Care & Handling of Basic Surgical Instruments-A Prospective. Int. J. Clin. Ski..

[12] Jackson S., Brady S. (2008). Counting difficulties: Retained instruments, sponges, and needles. AORN J..

[13] Nasir G.A.A. (2008). Missed Instrument and Surgical Sponge (Gauze and Pack). Internet J. Surg..

[14] Heibeyn J., König N., Domnik N., Schweizer M.R., Kinzius M., Janß A., Radermacher K. (2021). Design and Evaluation of a Novel Instrument Gripper for Handling of Surgical Instruments. Curr. Dir. Biomed. Eng..

[15] Toor J., Bhangu A., Wolfstadt J.I., Bassi G., Chung S., Rampersaud R., Mitchell W., Milner J., Koyle M. (2022). Optimizing the surgical instrument tray to immediately increase efficiency and lower costs in the operating room. Can. J. Surg..

[16] Ran B., Huang B., Liang S., Hou Y. (2023). Surgical Instrument Detection Algorithm Based on Improved YOLOv7x. Sensors.

[17] Ahmadi E., Masel D.T., Metcalf A.Y., Schuller K. (2019). Inventory management of surgical supplies and sterile instruments in hospitals: A literature review. Health Syst..

[18] Nast K., Swords K.A. (2019). Decreasing operating room costs via reduction of surgical instruments. J. Pediatr. Urol..

[19] Tracking Surgical Instruments with AI: A New Approach to Patient Safety. https://www.healthcareitnews.com/news/tracking-surgical-instruments-ai-new-approach-patient-safety.

[20] Yamazaki Y., Kanaji S., Matsuda T., Oshikiri T., Nakamura T., Suzuki S., Hiasa Y., Otake Y., Sato Y., Kakeji Y. (2020). Automated Surgical Instrument Detection from Laparoscopic Gastrectomy Video Images Using an Open Source Convolutional Neural Network Platform. J. Am. Coll. Surg..

[21] Sun Y., Pan B., Fu Y. (2022). Lightweight Deep Neural Network for Articulated Joint Detection of Surgical Instrument in Minimally Invasive Surgical Robot. J. Digit. Imaging.

[22] Nema S., Vachhani L. (2022). Surgical instrument detection and tracking technologies: Automating dataset labeling for surgical skill assessment. Front. Robot. AI.

[23] Wang Y., Sun Q., Liu Z., Gu L. (2021). Visual detection and tracking algorithms for minimally invasive surgical instruments: A comprehensive review of the state-of-the-art. Robot. Auton. Syst..

[24] Zhang B., Wang S.-S., Dong L., Chen P. (2020). Surgical Tools Detection Based on Modulated Anchoring Network in Laparoscopic Videos. IEEE Access.

[25] Deol E.S., Henning G., Basourakos S., Vasdev R.M., Sharma V., Kavoussi N.L., Karnes R.J., Leibovich B.C., Boorjian S.A., Khanna A. (2024). Artificial intelligence model for automated surgical instrument detection and counting: An experimental proof-of-concept study. Patient Saf. Surg..

[26] Wagner L., Schneider D.N., Mayer L., Jell A., Müller C., Lenz A., Knoll A., Wilhelm D. (2024). Towards multimodal graph neural networks for surgical instrument anticipation. Int. J. Comput. Assist. Radiol. Surg..

[27] Funke I., Mees S.T., Weitz J., Speidel S. (2019). Video-based surgical skill assessment using 3D convolutional neural networks. Int. J. Comput. Assist. Radiol. Surg..

[28] Lavanchy J.L., Zindel J., Kirtac K., Twick I., Hosgor E., Candinas D., Beldi G. (2021). Automation of surgical skill assessment using a three-stage machine learning algorithm. Sci. Rep..

[29] Namazi B., Sankaranarayanan G., Devarajan V. (2022). A contextual detector of surgical tools in laparoscopic videos using deep learning. Surg. Endosc..

[30] SAVI transforms surgical instrument tracking with Google Cloud | Google Cloud Blog. https://cloud.google.com/blog/products/ai-machine-learning/savi-transforms-surgical-instrument-tracking-with-google-cloud.

[31] Voros S., Long J.-A., Cinquin P. (2007). Automatic Detection of Instruments in Laparoscopic Images: A First Step Towards High-level Command of Robotic Endoscopic Holders. Int. J. Robot. Res..

[32] Neumuth T., Meissner C. (2012). Online recognition of surgical instruments by information fusion. Int. J. Comput. Assist. Radiol. Surg..

[33] ChatGPT. https://openai.com/chatgpt/overview/.

[34] OpenAI (2025). Hello GPT-4o. https://openai.com/index/hello-gpt-4o.

[35] Google (2024). Gemini. https://gemini.google.com/app/1018718432ef9dbf.

[36] SID by LayerJot. https://www.layerjot.com/sid.

[37] Schnock K.O., Biggs B., Fladger A., Bates D., Rozenblum R. (2017). Evaluating the Impact of Radio Frequency Identification Retained Surgical Instruments Tracking on Patient Safety: Literature Review. J. Patient Saf..

[38] Yamaguchi S., Soyama A., Ono S., Hamauzu S., Yamada M., Fukuda T., Hidaka M., Tsurumoto T., Uetani M., Eguchi S. (2021). Novel Computer-Aided Diagnosis Software for the Prevention of Retained Surgical Items. J. Am. Coll. Surg..

[39] Nadeau K. (2024). The Sterile Processing Department Digital Transformation. https://www.hpnonline.com/sterile-processing/article/53083618/the-sterile-processing-department-digital-transformation.

[40] Revolutionizing Sterile Processing Management with AI | CensisAI2. https://censis.com/solutions/ai2/.

[41] Kline A., Wang H., Li Y., Dennis S., Hutch M., Xu Z., Wang F., Cheng F., Luo Y. (2022). Multimodal machine learning in precision health: A scoping review. NPJ Digit. Med..

